# The subjective controllability of exotropia and its effect on surgical outcomes in patients with intermittent exotropia

**DOI:** 10.1186/s12886-023-02873-w

**Published:** 2023-03-28

**Authors:** Mirae Kim, Hong Kyun Kim, Won Jae Kim

**Affiliations:** 1 Nune Eye Hospital, Daegu, South Korea; 2 Department of Medicine, The Graduate School, Kyungpook National University, Daegu, South Korea; 3 Department of Ophthalmology, School of Medicine, Kyungpook National University, Daegu, South Korea; 4 Department of Ophthalmology, Yeungnam University College of Medicine, Daegu, South Korea

**Keywords:** Control, Exotropia, Surgical outcomes

## Abstract

**Background/aims:**

We evaluate the clinical characteristics of intermittent exotropia with controllability and compare surgical outcomes between patients with and without controllability.

**Methods:**

We reviewed the medical records of patients aged 6–18 years with intermittent exotropia who underwent surgery between September 2015 and September 2021. Controllability was defined as the patient’s subjective awareness of exotropia or diplopia associated with the presence of exotropia and ability to instinctively correct the ocular exodeviation. Surgical outcomes were compared between patients with and without controllability, with a favorable surgical outcome defined as an ocular deviation between ≤ 10 PD of exotropia and ≤ 4 PD of esotropia at distance and near.

**Results:**

Among 521 patients, 130 (25%, 130/521) had controllability. The mean age of onset (7.7 years) and surgery (9.9 years) were higher in patients with controllability than in those without controllability (p < 0.001). The mean control scores of patients with controllability (distance: 1.9, near: 1.5) were lower compared with patients without controllability (distance: 3.0, near: 2.2), reflecting a better level of control. Patients with controllability had a better surgical outcome than those without controllability, as analyzed by log-rank test (p < 0.001). Larger preoperative ocular exodeviation at distance (hazard ratio [HR] = 1.083, confidence interval [CI] = 1.018–1.151, p = 0.012) and near (HR = 1.102, CI = 1.037–1.172, p = 0.002) were significantly related to recurrence in patients with controllability.

**Conclusions:**

Patients with controllability showed better surgical outcomes, later exotropia onset, and better level of control than patients without controllability. Preoperative ocular exodeviation was a significant factor influencing favorable outcomes in patients with controllable exotropia.

## Background

Intermittent exotropia is the most common type of strabismus among Asians, particularly Koreans [1]. Exodrift and recurrence can occur after surgical treatment in these patients [2–4]. Previous studies have evaluated the clinical factors associated with surgical outcomes to predict the prognosis after surgical treatment [2–4]. The clinical factors mainly evaluated in patients with intermittent exotropia included the amount of ocular exodeviation, level of control, and results of stereoacuity tests.

Patients with intermittent exotropia present both objective findings and subjective symptoms associated with exotropia, such as light sensitivity, asthenopia, blurred vision, headache, and diplopia [5–8]. Among these subjective symptoms, some patients have a subjective awareness of the ocular exodeviation and can correct it instinctively; however, this often leads to asthenopia, diplopia, and headache [5, 8]. We defined these symptoms and ability to control exotropia as controllability and hypothesized that there might be an association between binocularity and the controllability of exotropia. While previous studies have evaluated surgical outcomes based on objective clinical factors, the association between surgical outcomes and subjective symptoms has rarely evaluated. This study evaluated the clinical characteristics of intermittent exotropia with controllability and compared surgical outcomes between patients with and without controllability.

## Methods

We retrospectively reviewed the medical records of pediatric patients aged 6–18 years with intermittent exotropia who underwent surgery between September 2015 and September 2021. Patients with basic type of intermittent exotropia with a distance deviation within 10 PD and near deviation were included. Controllability was defined as the patient’s subjective awareness of exotropia or diplopia associated with the presence of exotropia and an ability to instinctively correct the ocular exodeviation. Patients with any other type of strabismus, such as oblique muscle dysfunction, dissociated vertical deviation, A-V pattern, or nystagmus, were excluded from this study. Patients with previous intraocular surgery, neurological impairment such as cerebral palsy, or unilateral amblyopia were also excluded. Controllability was assessed by asking the patient directly about the presence of controllability at the initial visit and last examination before surgery. Patients with any disease such as autism spectrum disorder that made it difficult for them to communicate were also excluded as it was difficult to evaluate controllability. Patients with intermittent exotropia were divided into two groups according to the presence of controllability and the clinical characteristics and surgical outcomes were compared between the two groups. This study was approved by the institutional review board of *** University Hospital (IRB file number: 2022-01-055), which waived the requirement for informed consent owing to the retrospective nature of the study and the use of anonymized patient data.

### Preoperative evaluations

Exotropia onset was assessed based on patient’s or parental reports. The degree of deviation was measured using an alternate prism cover test at 6 m (distant fixation) and 33 cm (near fixation). An occlusive patch was provided to all patients at the first visit. All patients were informed that we planned to occlude the non-dominant eye for 1 h at the next visit to measure the largest exodeviation. The level of control was measured using the Look And Cover, then Ten seconds of Observation Scale for Exotropia (LACTOSE) control scoring system [9]. This control system was constructed by incorporating scales for both distance and near evaluations (5-point scales: 0–4 in each), yielding a total score ranging from 0 to 8. Higher and lower scores indicated poorer and better levels of control, respectively. More than three preoperative examinations were performed on each patient before the surgical treatment. Stereoacuity measurements were performed using the Lang I (LANG-STEREOTEST AG, Küsnacht, Switzerland) and Stereo Fly Stereotest (Stereo Optical Co., Chicago, IL, USA) in patients able to cooperate and complete the test.

### Surgical treatment and postoperative evaluations

All surgeries were performed under general anesthesia by a single surgeon (***). Conventional bilateral LR recession or unilateral R&R procedures for exotropia were performed using the surgical dose at the authors’ clinic (Table 1). In all patients, the operated muscles were reattached directly to the sclera without an adjustable suture. The angle of deviation measured on the day of surgery or 1 day after surgery was defined as the immediate postoperative deviation. All patients routinely used Tobradex (tobramycin/dexamethasone) eye drops four times daily and Effexin (ofloxacin) eye ointment once daily for 1 week postoperatively. The patients were followed up at 1, 3, 6, and 12 months after surgery and every 6 months thereafter. The postoperative angle of deviation was measured at each visit. Patients with at least 3 months of postoperative follow-up were included in the analysis. Favorable or successful surgical outcomes were defined as an ocular deviation in the primary position between ≤ 10 PD of exotropia and ≤ 4 PD of esotropia at distance and near. Recurrence was defined as exotropia > 10 PD at any time after surgery at distance or near deviation.

**Table 1:** Surgical dose of bilateral lateral rectus recession and unilateral lateral rectus recession and medial rectus resection

Prism diopters	Bilateral LR recession	Unilateral LR recession and MR resection	
	Recession amount of LR (millimeter)	Recession amounts of LR (millimeter)	Resection amounts of MR (millimeter)
20	5/5	-	-
25	6/6	4	3
30	-	4	4
35	-	5	4
40	-	5	5
45	-	7	5
50	-	8	5

LR, Lateral rectus muscle; MR, medial rectus muscle

### Statistical analysis

Statistical analyses were performed using IBM SPSS Statistics for Windows, version 20.0 (IBM Corp., Armonk, NY, USA). Unpaired t- and chi-square tests were used to evaluate differences between patients with and without controllability. The cumulative probabilities of success were assessed according to Kaplan–Meier life-table analysis. Log-rank tests were used to compare survival rates between the two groups. Cox proportional hazards regression analysis was used to identify the risk factors associated with recurrence after surgery in patients with controllability. Statistical significance was set at p < 0.05.

## Results

This study included 521 patients (271 male, 250 female) with intermittent exotropia, 130 (25%, 130/521) of whom had controllability. The basic characteristics of the patients with intermittent exotropia with and without controllability are shown in Table 2. Sex, amount of ocular exodeviation, surgical methods, and results of stereoacuity tests did not differ between patients with and without controllability. The mean ages of exotropia onset (7.7 years) and surgery (9.9 years) were higher in patients with controllability than in those without controllability (5.6 years and 8.3 years, respectively). The mean control scores at distance, near, and overall of patients with controllability were 1.9, 1.5, and 3.4, respectively. The control scores for distance, near, and overall were lower in patients with controllability, reflecting a better level of control, compared to patients without controllability (mean distance, near, and overall control scores were 3.0, 2.2, and 5.2, respectively, all p < 0.001). Patients with controllability were more myopic than patients without controllability, probably because patients often experience myopic changes with age.

**Table 2:** Comparison of basic characteristics between patients with and without controllability of exotropia

N = 521	With controllability (N = 130)	Without controllability (N = 391)	p-value
Sex (Male: Female)	58: 72	213: 178	0.051
Mean onset of exotropia (range), yr	7.7 ± 2.8 (107/130, 2–17)	5.6 ± 3.0 (334/391, 0–18)	< 0.001
Preoperative ocular exodeviation (range), PD			
Distance	27.2 ± 7.2 (16–50)	26.8 ± 6.7 (16–50)	0.581
Near	31.0 ± 7.4 (16–55)	30.0 ± 6.8 (18–55)	0.168
Level of control, control score	109/130	282/391	
Distance	1.9 ± 1.1	3.0 ± 0.9	< 0.001
Near	1.5 ± 0.8	2.2 ± 1.1	< 0.001
Overall	3.4 ± 1.7	5.2 ± 1.7	< 0.001
Spherical equivalent refractive errors, D			
Right eye	-1.72 ± 1.60 (-7.25 to + 1.00)	-1.09 ± 1.55 (-7.00 to + 4.50)	< 0.001
Left eye	-1.80 ± 1.61 (-8.00 to + 2.00)	-1.08 ± 1.58 (-7.50 to + 5.00)	< 0.001
Mean age of surgery (range), yr	9.9 ± 8.3 (6–18)	8.3 ± 2.4 (6–18)	< 0.001
Surgical methods			
Bilateral LR recession	73	232	
Unilateral R&R	57	159	0.524
Results of stereotest			
Lang I test, passed, (%)	127 /128 (99.2)	353 /366 (96.4)	0.104
Stereo Fly Stereotest (≤ 100 arcsec, %)	115 /128 (89.8)	311 /361 (86.1)	0.284

PD: prism diopters; D: diopters; LR: lateral rectus; R&R: lateral rectus recession and medial rectus resection; arcsec: arcsecond

The level of control was measured using the Look And Cover, then Ten seconds of Observation Scale for Exotropia (LACTOSE) control scoring system. This control system was constructed by incorporating scales for both distance and near evaluations (5-point scales: 0–4 in each), yielding a total score ranging from 0 to 8. Higher and lower scores indicated poorer and better levels of control, respectively

### Surgical outcomes in patients with and without controllability of exotropia

**Fig. 1:**
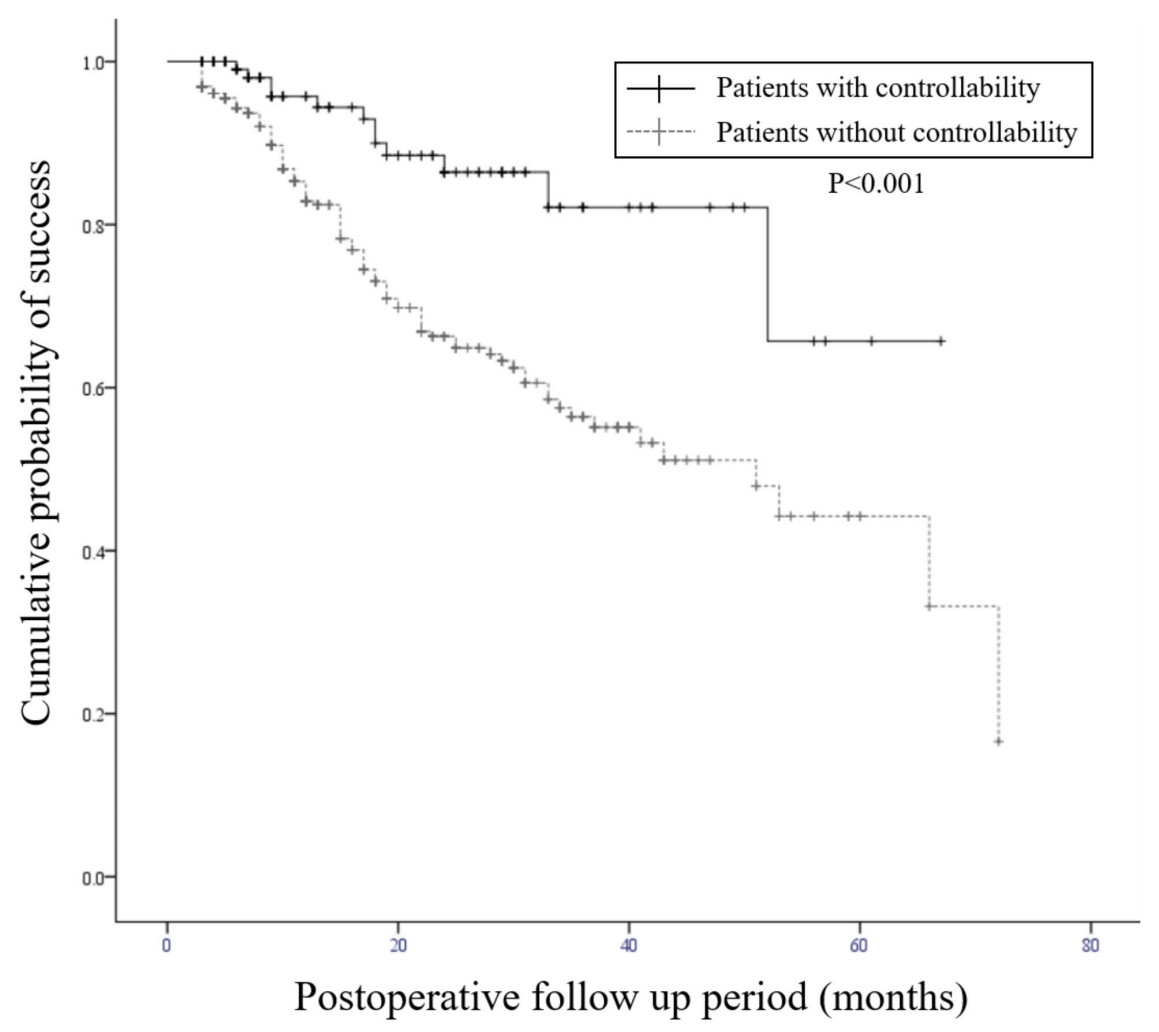
Comparison of Kaplan–Meier survival curves of surgical outcomes between patients with and without exotropia controllability. The recurrence-free survival curve differs between the groups (p < 0.001, log-rank test). The cumulative probability of success considering recurrence as the event in patients with controllability were 94.4%, 86.4%, 82.1%, and 65.7% at 1, 2, 3, and 4 years after surgery, respectively. In contrast, the probabilities of patients without controllability were 82.9%, 66.3%, 56.4%, and 51.1%, respectively

The immediate postoperative ocular exodeviation was − 3.6 ± 1.9 PD at distance and − 0.3 ± 2.6 PD at near in patients with controllability, in which negative and positive numbers indicate esodeviation and exodeviation, respectively. Patients without controllability showed − 3.5 ± 2.4 PD at distance and − 0.3 ± 2.6 PD at near immediately after surgery, with no significant difference between the two groups (p = 0.594 at distance, p = 0.987 at near). Kaplan–Meier survival analysis showed cumulative probability of success rates considering recurrence as the event in patients with controllability of 94.4%, 86.4%, 82.1%, and 65.7% at 1, 2, 3, and 4 years after surgery, respectively. In contrast, the rates in patients without controllability were 82.9%, 66.3%, 56.4%, and 51.1%, respectively. The patients with controllability had a better recurrence-free survival curve than patients without controllability, as analyzed by log-rank test (p < 0.001, Fig. 1).

### Factors associated with recurrence in patients with controllability

Among patients with controllability, the clinical factors associated with recurrence were evaluated using Cox proportional hazards regression analysis. Larger preoperative ocular exodeviation at distance (HR = 1.083, CI = 1.018–1.151, p = 0.012) and near (HR = 1.102, CI = 1.037–1.172, p = 0.002) were significantly associated with recurrence in patients with exotropia and controllability (Table 3).

**Table 3:** Cox proportional hazard regression analysis for recurrence of exotropia in patients with controllability

N = 130	HR	95% CI	p-value
Sex	1.092	0.344–3.472	0.881
Mean onset of exotropia	0.992	0.815–1.208	0.938
Preoperative ocular exodeviation			
Distant	1.083	1.018–1.151	0.012
Near	1.102	1.037–1.172	0.002
Level of control, Control score			
Distant	1.037	0.599–1.797	0.897
Near	1.701	0.914–3.166	0.094
Overall	1.176	0.835–1.657	0.354
Spherical equivalent refractive errors			
Right eye	1.232	0.816–1.861	0.322
Left eye	1.039	0.704–1.533	0.848
Mean age of surgery (range), yr	0.891	0.685–1.158	0.388
Immediate postoperative ocular deviation			
Distant	0.985	0.784–1.239	0.898
Near	1.073	0.866–1.329	0.519

The level of control was measured using the Look And Cover, then Ten seconds of Observation Scale for Exotropia (LACTOSE) control scoring system. This control system was constructed by incorporating scales for both distance and near evaluations (5-point scales: 0–4 in each), yielding a total score ranging from 0 to 8. Higher and lower scores indicated poorer and better levels of control, respectively

## Discussion

In this study, patients with controllability had a later onset of exotropia and a better level of control than patients without controllability. Patients with controllability had better surgical outcomes than patients without controllability. Preoperative ocular exodeviation was a significant factor influencing favorable outcomes in patients with exotropia and controllability.

The subjective symptoms associated with exotropia may differ among patients with intermittent exotropia [5–7]. The fusional potential and subjective symptoms of exotropia may be associated. von Nooden observed that the clinical course of exotropia differed depending on the state of the sensorimotor system [5]. For example, a child with large exophoria without any symptoms may develop exotropia in later life or stay exophoric and develop symptoms of eyestrain with sustained close work. We were interested in the controllability of exotropia and evaluated the association between surgical outcomes and controllability.

In this study, 25% of patients reported exotropia controllability. The degree of ocular exodeviation was variable in these patients. Even patients with a large amount of deviation (> 50 PD) showed controllability. A previous study reported that 42% of children expressed a general awareness of exodeviation and various ocular sensations [9]. In their study, some children also demonstrated awareness of their ability to correct the exodeviation by blinking.

The surgical outcomes in patients with controllability were better than those in patients without controllability. Preoperative and immediate postoperative ocular alignment did not differ significantly between patients with and without controllability. Therefore, the differences in surgical outcomes were not due to the immediate effects of surgical treatment. The level of control using LACTOSE control scoring system is better in patients with controllability. The better surgical outcomes in patients with controllability may be due to better binocularity, as patients with controllability may have better binocularity than those without. These results may be consistent with previous study by Moon et al. that higher distance and near LACTOSE scores representing worse control of deviation were associated with higher rates of surgical failure in children with intermittent exotropia [10].

Patients with controllability showed a relatively later onset of exotropia than those without controllability, possibly because patients with better binocularity can maintain normal ocular alignment for a longer time, leading to older mean ages of onset and surgery. Similarly, patients with better binocularity may hide the total amount of ocular exodeviation, leading to variability in ocular exodeviation during follow-up [11–13]. Lim and Kim showed a higher possibility of dramatic decreases in ocular alignment before impending surgery caused by anxiety in pediatric patients with controllability [12]. These patients had relatively better level of control [12]. Lee et al. reported better surgical outcomes of exotropia in patients with increased ocular deviation after the monocular occlusion test [13]. They postulated that these patients had a better preoperative fusion rate, which may have concealed the total amount of ocular deviation before the occlusion test. Their better potential fusion capacity may have influenced their response to surgery and facilitated stable surgical outcomes [13].

The decision to agree to surgery in pediatric patients with a large amount of exotropia but good control may be difficult for parents. Parental observations are more likely to correlate with the level of control than with the amount of ocular deviation in pediatric patients with intermittent exotropia [14]. Patients with controllability usually have a good level of control, with variable amounts of ocular deviation. Therefore, parents cannot easily notice ocular deviation and may hesitate to agree to surgery in patients with controllability. Not all patients with exotropia and controllability require surgical treatment [6]. We recommended surgery in patients presenting with any symptoms associated with difficulty on control of exotropia, including asthenopia, diplopia, and headache, or if the trend of these symptoms increases with age. The exotropia gradually progresses with age [15–17]. Surgical outcomes are more favorable in patients with controllability than in those without. The results of this study suggest that surgical treatment may be more appropriate in patients with exotropia and controllability.

Our results may help predict prognosis after surgery and may also provide data for the creation of a new classification system for patients with intermittent exotropia. The current general classification system for exotropia is based on the main difference between ocular exodeviation and distant and near fixation or level of control [5, 6]. While these systems are useful in the clinical setting, there may be limitations in predicting the surgical results. Future classification systems that consider not only clinical findings, but also subjective symptoms may be appropriate to better understand exotropia and predict surgical outcomes.

In patients with controllability, a larger preoperative amount of deviation at distance and near was significantly associated with recurrence. These results are consistent with those of previous studies on the prognostic factors of surgical outcomes in patients with intermittent exotropia [2–4]. Although patients with controllability had relatively better binocularity, those with a large amount of exotropia were more likely to experience recurrence after surgical treatment.

This study had several limitations. First, we did not evaluate the changes in controllability and subjective awareness of exotropia after surgery. Ha and Kim showed improved subjective symptoms including stereopsis and asthenopia after surgery in patients with constant exotropia [18]. We evaluated only the association between surgical outcomes and the presence of controllability. The changes in controllability and awareness of exotropia require assessment in future studies. Second, this study included only pediatric patients with intermittent exotropia. The clinical characteristics of adult and pediatric patients with intermittent exotropia may differ [19]. Third, the presence of controllability was assessed by directly asking the patient and each patient’s specific accompanying symptoms did not investigate in this study. Further study using questionnaire to evaluate the controllability and accompanying symptoms will be performed.

## Conclusion

In conclusion, about a quarter of pediatric patients with intermittent exotropia had subjective controllability of exotropia. Patients with controllability showed better surgical outcomes, later exotropia onset, and better level of control than patients without controllability. Preoperative ocular exodeviation was a significant factor influencing favorable outcomes in patients with controllable exotropia.

## Data Availability

All data generated or analysed during this study are included in the manuscript.

## List of abbreviations

PD: prism diopters
LR: lateral rectus muscle
R&R: lateral rectus muscle recession and medial rectus muscle resection
